# Multiplex LAMP assay for detecting the prevalent species of dust mites *Dermatophagoides farinae* and *Dermatophagoides pteronyssinus* in the domestic environment

**DOI:** 10.1038/s41598-024-66043-8

**Published:** 2024-07-12

**Authors:** Yujun Shuai, Qiqi Xue, Huanxin Tu, Junjie Guo, Qiao Teng, Yueye Xu, Jingang Xu, Yuanyuan Li, Hongming Zhou, Jinhong Zhao

**Affiliations:** 1 Department of Medical Parasitology, Wannan Medical College, Wuhu, 241002 Anhui China; 2 Department of Laboratory Medicine, Third Affiliated Hospital of Naval Medical University, Shanghai, 200438 China; 3 Department of Medical Parasitology, Qiqihaer Medical College, Qiqihaer, 161000 Heilongjiang China; 4 Anhui Province Key Laboratory of Biological Macro-molecules Research, Wannan Medical College, Wuhu, 241002 Anhui China

**Keywords:** Multiplex LAMP assay, PCR, House dust mites, Visual detection, *Dermatophagoides farinae*, *Dermatophagoides pteronyssinus*, Biological techniques, Medical research

## Abstract

*Dermatophagoides farina* (*D. farinae*) and *Dermatophagoides pteronyssinus* (*D. pteronyssinus*) are the prevalent kinds of house dust mites (HDMs). HDMs are common inhalant allergens that cause a range of allergic diseases, such as rhinitis, atopic dermatitis, and asthma. The epidemiology of these diseases is associated with exposure to mites. Therefore, in the present study, a method named multiplex loop-mediated isothermal amplification (LAMP) was developed to detect environmental dust mites. The multiplex LAMP assay allows amplification within a single tube and has an ITS plasmid detection limit as low as 40 fg/µL for both single dust mites and mixed dust mites (*D. pteronyssinus* and *D. farinae*), which is up to ten times more sensitive than classical PCR techniques. Furthermore, the multiplex LAMP method was applied to samples of single dust mites and clinical dust to confirm its validity. The multiplex LAMP assay exhibited higher sensitivity, simpler instrumentation, and visualization of test results, indicating that this method could be used as an alternative to traditional techniques for the detection of HDMs.

## Introduction

House dust mites (HDMs) have been named for their inhabitation of domestic dust. HDMs readily obtain food from the skin debris that people in daily. *Dermatophagoides farinae* (*D. farinae*) and *Dermatophagoides pteronyssinus* (*D. pteronyssinus*) are prevalent and widespread among all HDMs^1^. Dust mite allergens, including eggs, feces, bodies, and corpse residues, are common allergens and might cause allergic rhinitis, atopic dermatitis, and allergic asthma^2–4^. According to previous reports, approximately 10% of the average public and over 80% of allergic asthmatics undergo anaphylactic reactions when exposed to HDMs^5^. In the Asia–Pacific region of allergic diseases, HDMs have been reported to be one of the major allergens involved in allergic rhinitis^6^. In Hong Kong, sensitivity to HDMs is prevalent in more than 80% of children diagnosed with asthma^7^. Amin et al.^8^ investigated the prevalence of sensitivity to aeroallergens in patients with atopic dermatitis and reported that patients exhibited greater sensitivity to *D. pteronyssinus* (50%) and *D. farinae* (34%) than to birch pollen, cat epithelium, and cockroaches. Therefore, it is crucial to detect these mites using multiple diagnostic tests with high specificity, sensitivity, and simultaneous detection.

HDMs can be detected by morphological, immunological, and molecular biology approaches^9–12^. Morphological detection is a low-cost method that mostly involves the detection of HDMs under a microscope in the laboratory. However, HDMs tend to hide in dust and are tiny. Therefore, morphological tests for the detection of HDMs are time-consuming and prone to missed detection^1,2^. Immunological studies have shown that the amino acid sequences of the *D. pteronyssinus* allergen Der p 1 and *D. farinae* allergen Der f 1 are extremely similar, up to 83%; thus, there is a large degree of homology, and cross-reactivity between these allergens is present^13,14^. Molecular biological methods with high specificity and sensitivity have been widely used for the diagnosis of allergic diseases. In molecular biology, PCR is one of the most classical and representative amplification techniques. Yang et al.^15^ previously used PCR to taxonomically identify six distinct mite types. Oliveira et al.^12^ established a multiplex PCR for HDMs. The above methods, however, are time-consuming and cumbersome. They also require costly laboratory equipment, dedicated laboratory facilities, and trained staff^16^.

**Figure 1:**
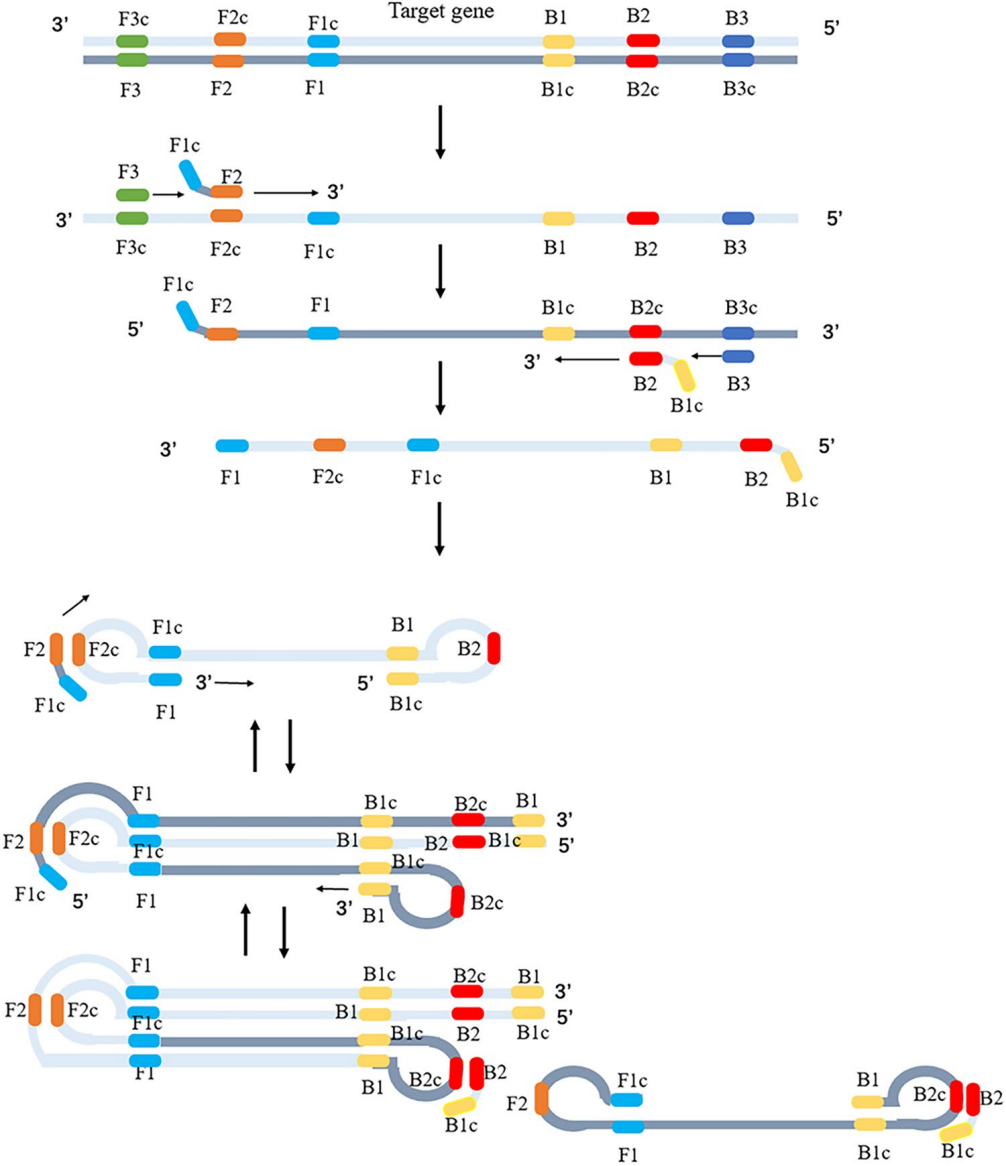
Schematic representation of the LAMP reaction. DNA synthesis relied on DNA polymerase with high strand substitution activity, two outer primers (F3 and B3), and a set of two specially designed inner primers (FIP and BIP). When FIP (5’-F1c-F2) combines with F2c, complementary strand synthesis is initiated. When F3 combines with F3c, strand-substituted DNA synthesis is initiated, which releases the FIP-linked complementary strand that is used in single-stranded DNA. BIP (5’-B1c-B2) initiates strand displacement DNA synthesis, and subsequently, B3 initiates strand-substituted DNA synthesis. This leads to the production of dumbbell-shaped DNA, which serves as the foundation for subsequent reactions. This single-stranded DNA is used for BIP-initiated DNA synthesis and subsequent B3-primed strand-replacement DNA synthesis, resulting in the production of dumbbell-shaped DNA. This dumbbell-shaped DNA then serves as the foundation for the LAMP cycle, which proceeds continuously through chain substitution reactions with the inner primer cycle, repetitively and spontaneously, yielding amplification products of different lengths. This schematic illustrates only the single-target response mechanism.

Loop-mediated isothermal amplification (LAMP) has been successfully carried out in diverse fields of study, such as for ABO genotyping, *Salmonella*, severe acute respiratory syndrome coronavirus 2 (SARS-CoV-2), *Toxoplasma gondii*, and shrimp allergens, in a rapid, highly efficient and low-detection-limit manner compared to conventional PCR^17–21^. LAMP technology was reported by Notomi et al. in 2000^22^. This amplification methodology involves the addition of four to six specific primers that are capable of recognizing up to six to eight fragment sequences on the target gene within the same reaction (Fig. 1), which is different from classical PCR amplification techniques. LAMP technology also uses a thermostable polymerase, *Bst-*DNA polymerase, with a high displacement capacity, in which the second strand is displaced without thermal denaturation during the reaction. The reaction can be completed within 30–60 min under constant temperature conditions (60–68 °C) and can be performed without a DNA amplicon. The specificity of the method is a result of the use of four or six primers in the reaction process^22,23^. The amplification reaction products generated at the reaction endpoint may be detected using various methods, including gel electrophoresis, measurement of the turbidity derived from magnesium pyrophosphate precipitation, or the addition of fluorescent chemicals to the reaction compounds for visual observation^24,25^. To increase detection efficiency and decrease experimental costs, several researchers have used LAMP technology to detect multiple target genes of the same pathogen or screen multiple pathogens^26,27^. When using multiplex LAMP for detection, cross-reaction between primers must be prevented, which necessitates designing multiple complex primers in advance. Kumar et al.^28^ combined the LAMP technique with magnetic particle visualization for the detection of *chikungunya* and *dengue* viruses. Joshi et al.^29^ established a multiplex LAMP method for the differential diagnosis of *Leishmania donovani* and *Mycobacterium leprae* by means of a real-time fluorescence analyzer. Wang et al.^26^ used real-time turbidimetry to establish multiplex LAMP for the primary screening of *Perkinsus* and *Bonamia* infections in shellfish parasites present in aquatic products.

We aimed to establish a multiplex LAMP method to increase the detection efficiency and decrease the experimental cost of *D. pteronyssinus* and *D. farinae*. The experimental results were illustrated using real-time turbidity curves and fluorescence detection reagents (FDRs). The developed method is expected to become popular in the field of point-of-care detection and on-site diagnosis of dust mites present in the environment as allergens owing to its rapid, efficient, and highly specific qualities and the simplicity of the equipment required for detection.

## Materials and methods

### Sample collection and DNA extraction

Dust mites were obtained from floor dust, air-conditioned filter dust, and bed dust from a student dormitory of Wannan Medical College (Anhui, China). The samples were examined under a light microscope for preliminary morphological identification of individual mites. *D. farinae* was cultured in the laboratory of Wannan Medical College (Anhui, China) in an incubator at 25 ± 2 °C and 70% ± 5% RH.

Specificity testing was conducted using mites belonging to other mite species, *Cheyletus malaccensis* (*C. malaccensis*), *Scheloribates laevigatus* (*S. laevigatus*), *Aleuroglyphus ovatus* (*A. ovatus*) and *Lepidoglyphus destructor* (*L. desctructor*), and other organisms commonly present in living environment dust, *Musca domestica* (*M. domestica*), *Anopheles sinensis* (*An. sinensis*), and *Periplaneta americana* (*P. americana*). The above samples were deposited in the Department of Medical Parasitology, Wannan Medical College, Anhui Province, China.

For all experimental samples, genomic DNA was extracted following the procedure provided by the manufacturer of the DNeasy Blood and Tissue Kit (Tiangen Biotech Beijing Co. Ltd., China). Moreover, for the dust samples collected, genomic DNA was extracted following the procedure provided by the manufacturer of the TIANamp Soil DNA Kit (Tiangen Biotech Beijing Co. Ltd., China). The above extracted genomic DNA samples were stored at − 20 °C until use.

### Primer design

The internal transcribed spacer (ITS) region of conserved rDNA sequences has a certain degree of interspecies specificity and intraspecies conservation and is therefore used as the target point for interspecies identification. Therefore, the ITS gene was selected as the target gene in this study, and the ITS sequence of *D. pteronyssinus* (KC215340) was downloaded from the NCBI GenBank database. Five sets of LAMP candidate primer sets against the different regions of the target gene were designed online and automatically using Primer Explorer V5 (http://primerexplorer.jp/e/) (Eiken Chemical Co., Tokyo, Japan). The primers used for *D. farinae* were previously reported by Xue et al.^30^. All primers in our study were synthesized at Sangon Biotechnology (Sangon Biotech Shanghai Co. Ltd., China) and later diluted following the instructions provided by the manufacturer. Finally, the diluted primers were stored at − 20 °C until use.

### Establishment of a single LAMP assay

Each LAMP reaction system was 25 µL, which contained 12.5 µL of 2 × LAMP reaction buffer (including 0.1% Tween 20, 20 mM Tris–HCl (pH 8.8), 10 mM (NH_4_)_2_SO_4_, 10 mM KCl, 0.8 M betaine, 8 mM MgSO_4_, and 1.4 mM dNTPs), 1 µL of *Bst*-DNA polymerase (8 U), 1 µL of FDRs (LAMP DNA Amplification Kit; Eiken Chemical Co. Ltd., Tochigi, Japan), and 2 µL of DNA template. All of the above reagent compositions were consistent during the configuration of the LAMP reaction system for *D. pteronyssinus* and *D. farinae*. While the single LAMP primer volume of *D. pteronyssinus* was 4 µL (40 µM FIP and BIP, 5 µM F3 and R3), the single LAMP primer volume of *D. farinae* was 5 µL (5 µM F3 and R3, 20 µM LB, 40 µM FIP and BIP). A negative control was selected for the DNAzyme-free water.

Primer screening was conducted using five sets of primers designed for *D. pteronyssinus* DNA, which were initially amplified using a 65 °C heating block for 60 min and then enzymatically extinguished at 80 °C for 10 min. The results were visualized through real-time turbidity formation curves and FDRs. A suitable temperature favors stimulation by *Bst*-DNA polymerase activity.

Temperature screening was conducted by incubating the reaction at a temperature of 62 °C to 69 °C (1 °C as a gradient) for 60 min, after which the reaction was terminated by incubation at 80 °C for 10 min. The single LAMP reaction conditions for *D. farinae* used in the present study were designed previously in our laboratory, as reported by Xue et al.^30^.

The specificity of the single LAMP method was then assessed using the ITS plasmids of *D. pteronyssinus* and *D. farinae* as positive controls. The genomic DNA templates of *A. ovatus*, *C. malaccensis*, *L. destructor*, *S. laevigatus*, *P. americana*, *An. sinensis*, and* M. domestica* were used as specific control samples. A negative control was selected for the DNAzyme-free water. The sensitivity of the single LAMP method was assessed by preparing ten-fold dilutions of the ITS plasmids of both species in the concentration range of 40 ng/µL to 400 ag/µL and then using these plasmids as templates for the assay.

### Establishment of the multiplex LAMP assay

The best primers selected from the single LAMP reaction system, as described above, were assembled into the multiplex LAMP reaction system to establish the multiplex LAMP assay. Each LAMP reaction mixture had a volume of 25 µL, which contained 12.5 µL of two-fold LAMP reaction buffer, 1 µL of *Bst*-DNA polymerase (8 U), 1 µL of FDRs, 3.5 µL of the primer for *D. farinae* (10 µM F3 and R3, 40 µM FIP and BIP, 40 µM LB), 3 µL of the primer for *D. pteronyssinus* (10 µM F3 and R3, 40 µM FIP and BIP), 2 µL of the positive plasmid template (1 µL for *D. pteronyssinus* and 1 µL for *D. farinae*), and the remaining volume of the DNAzyme-free water. A negative control was selected for the DNAzyme-free water.

### Detection of the LAMP products

**Figure 2:**
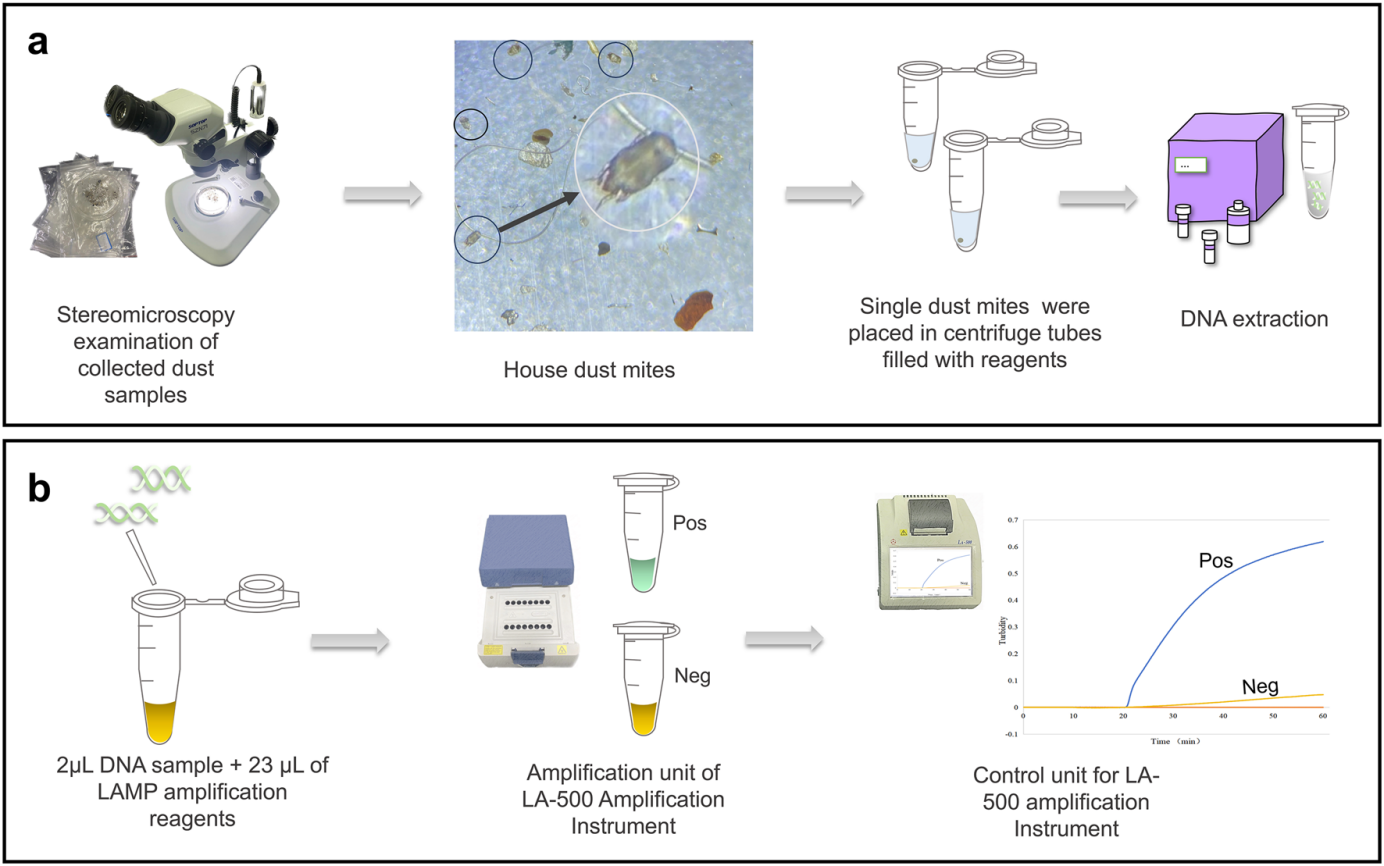
The image is an outline of this study. (**a**) Outline illustrations of sample preparation and DNA extraction. (**b**) Outline illustrations of LAMP detection. Amplification unit: a positive reaction is depicted in fluorescent green, and a negative reaction is depicted in fluorescent orange with no discolouration. Control unit: A positive sample amplification produces significant amounts of insoluble magnesium pyrophosphate precipitate, and the turbidity profile (x-axis is time, y-axis is turbidity) is displayed on the operating panel of the control unit.

The LAMP amplification results were calibrated for 6 s using a loopamp real-time turbidimeter (LA-500; Eken Chemical Co. Ltd., Tochigi, Japan). The reaction results obtained with the Loopamp real-time turbidimeter were standardized using a positive curve. At the same time, to allow immediate visualization, the results were visually inspected by adding 1 µL of FDRs (Eiken Chemical Co. Ltd., Tochigi, Japan) to the reaction system, followed by observing the color change in the reaction samples (Fig. 2). Positive and negative LAMP products were defined as fluorescent green and fluorescent orange signals, respectively, as observed by the naked eye under natural light.

### Evaluation of multiplex LAMP specificity

The specificity of the multiplex LAMP detection method was verified using the ITS plasmids of *D. pteronyssinus*, *D. farinae*, *D. pteronyssinus*, and *D. farinae* as positive controls. The genomic DNA samples of *A. ovatus, C. malaccensis, L. destructor, S. laevigatus, P. americana, An. sinensis, and M. domestica* were used as specific control samples. A negative control was selected for the DNAzyme-free water. The results were validated by repeating the assessment three times for each sample concentration.

### Evaluation of multiplex LAMP sensitivity

The sensitivity and LODs of the multiplex LAMP assay were obtained by mixing equal volumes of the same concentrations of the two plasmids. The ITS plasmids of *D. pteronyssinus*, *D. farinae* and *D. pteronyssinus* and *D. farinae* were serially diluted to prepare different concentrations (40 ng/µL, 4 ng/µL, 400 pg/µL, 40 pg/µL, 4 pg/µL, 400 fg/µL, 40 fg/µL, 4 fg/µL, and 400 ag/µL). The results were validated by repeating the assessment three times for each sample concentration. A negative control was selected for the DNAzyme-free water.

### Detection of clinical samples

The effectiveness of the multiplex LAMP assay was determined by examining the detection of a single *D. pteronyssinus* sample, a single *D. farinae* sample, and a mixed single mite sample (*D. pteronyssinus* and *D. farinae*). In each group, ten samples were prepared for testing. Mite DNA was extracted according to the instructions of the DNeasy Blood & Tissue Kit (Tiangen Biotech Beijing Co. Ltd., China). The effectiveness of the multiplex LAMP method and the PCR method was detected for all samples.

A total of 55 floor dust, air-conditioned filters and bed dust samples collected from the student dormitories were collected in November 2023 and December 2023. The floor dust and air-conditioned filter dust were collected by a small brush, and a bed brush was used to pat down the bed surfaces and bed mattresses to collect dust. All collected samples were discarded for visible large particles of debris or hair and then placed under a stereomicroscope for observation and classification. DNA was extracted from the samples using the TIANamp Soil DNA Kit (Tiangen Biotech Beijing Co. Ltd., China) according to the manufacturer’s instructions. All samples were detected by the multiplex LAMP method, after which the percentage of positive samples was determined. IBM SPSS statistical software version 27 (SPSS Inc., Chicago, IL) was used for the statistical analysis of the results, and χ^2^ value and P-value were determined to reveal significant differences.

### PCR assay

Comparison of clinical sample specificity, sensitivity and detection efficacy using PCR assays. The PCR system had a volume of 25 µL, which contained 12.5 µL of the PrimeSTAR Max DNA Polymerase (8 mM Mg^2+^ and 0.4 mM each for dNTPs) (Takara Biomedical Technology Co. Ltd., Beijing, China), 2 µL of the primer for *D. farinae* (10 µM F3 and R3), 2 µL of the primer for *D. pteronyssinus* (10 µM F3 and R3) 1 µL each of F3 and B3 (10 µM), and 2 µL of the DNA template, with the remaining volume made up of the DNAzyme-free water. The primers used were the F3 primer and B3 primer. The cycling conditions were as follows: initial 5 min at 94 °C; 35 cycles of 15 s at 94 °C, 30 s at 55 °C, and 30 s at 72 °C; and final 5 min at 72 °C. The PCR products were then subjected to electrophoresis on a 2% agarose gel, followed by staining with 4S Red Plus nucleic acid stain (Sangon Biotech Shanghai Co. Ltd., China). Images were analyzed semiquantitatively using a Bio-Rad Gel Doc EQ imaging system (Bio-Rad, Hercules, CA, USA).

## Results

### Optimization of the single LAMP assay

**Figure 3:**
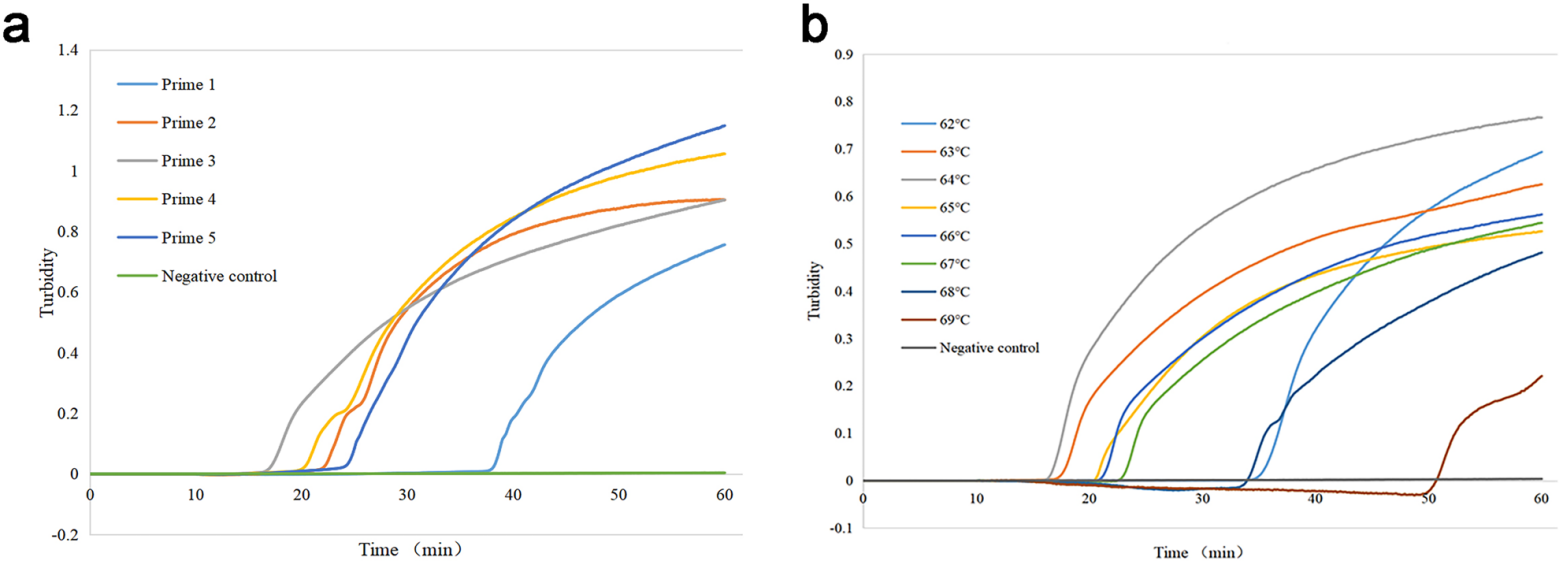
Optimization of the reaction conditions for the single LAMP assay for *D. pteronyssinus*. A negative control was selected for the DNAzyme-free water. (**a**) Screening of the designed 5 primer pairs. The primer screening results revealed that the 4th primer pair had the highest amplification efficiency. (**b**) Reaction temperature optimization: Eight different ladders were tested at different temperatures—62 °C, 63 °C, 64 °C, 65 °C, 66 °C, 67 °C, 68 °C, and 69 °C—and 64 °C was identified as the optimal temperature. The results were observed within 60 min of the reaction.

**Figure 4:**
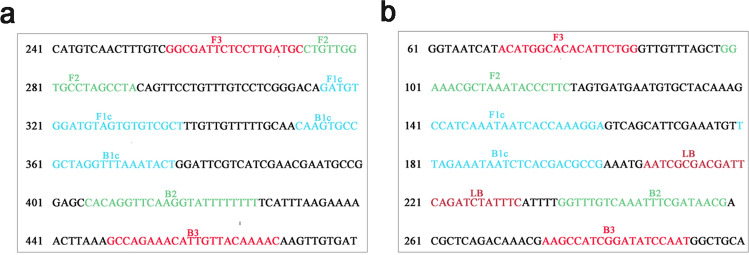
Schematic representation of primer positions. (**a**) *D. pteronyssinus* ITS sequence (GenBank accession number KC215340). (**b**) *D. farinae* ITS sequence (GenBank accession number MH793957).

The optimal primers for *D. pteronyssinus* were determined by conducting LAMP reactions using different sets of primers. Amplification was performed at 65 °C for 60 min, after which the turbidity of the LAMP products was measured. Although each primer set produced amplification product curves with significant turbidity, the amplification efficiency of each primer differed. In the turbidity analysis, primer 3 appeared to be the fastest, but it also amplified *D. farinae* significantly (Supplementary Fig. S1 online). Therefore, the primer with the second highest amplification efficiency, primer 4, was selected as the optimal primer for *D. pteronyssinus*. Next, the reaction temperature was optimized. The reaction amplification curves revealed that the best amplification efficiency was achieved at 64 °C (Fig. 3). Therefore, 64 °C was selected as the optimal reaction temperature for subsequent experiments. In the case of *D. farinae*, the report by Xue et al.^30^ was referred to for selecting the optimum primer (primer 4) and temperature (64 °C). The sequences and positions of the optimal primers determined through optimization experiments are presented in Table 1 and Fig. 4.

**Table 1:** LAMP primer sequences for ITS genes of *D. pteronyssinus and D. farinae.*

Gene Name	Primers	Sequence (5′-3′)	References
ITS of *D. pteronyssinus*	F3	GGCGATTCTCCTTGATGC	
	B3	GTTTTGTAACAATGTTTCTGGC	This study
	FIP	AGCGACACACTACATCCACATCCTGTTGGTGCCTAGCCTA	
	BIP	CAAGTGCCGCTAGGTTTAAATACTAAAAAAAATACCTTGAACCTGTG	
ITS of *D. farinae*	F3	ACATGGCACACATTCTGG	
	B3	ATTGGATATCCGATGGCTT	^30^
	FIP	TCCTTTGGTGATTATTTGATGGCTGGAAACGCTAAATACCCTTC	
	BIP	TTAGAAATAATCTCACGACGCCGCGTTATCGAAATTTGACAAACC	
	LB	AATCGCGACGATTCAGATCTATTTCAATCGCGACGATTCAGATCTATTTC

### Specificity and sensitivity of the single LAMP assay

**Figure 5:**
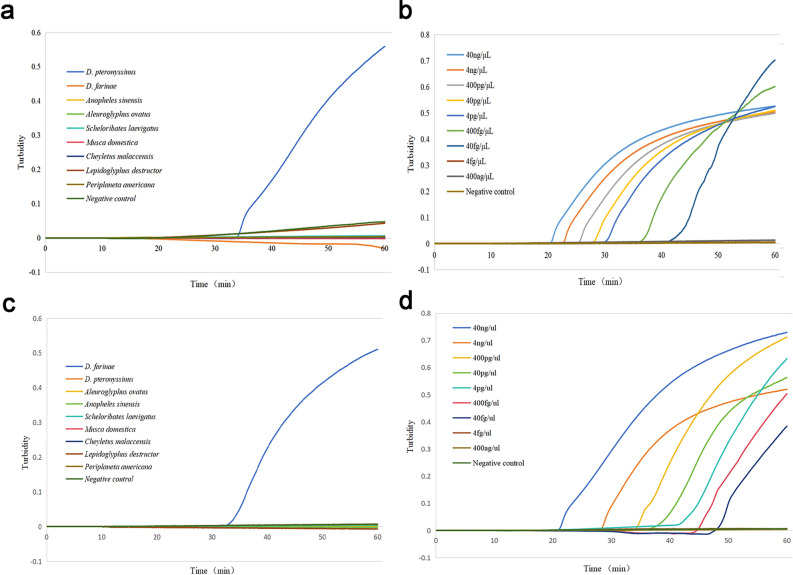
Real-time turbidity formation curves obtained for the single LAMP assays. (**a**) Specificity assay conducted for *D. pteronyssinus* alone. (**b**) Sensitivity assay conducted for *D. pteronyssinus* alone. (**c**) Specificity assay conducted for *D. farinae* alone. (**d**) Sensitivity assay conducted for *D. farinae* alone.

Optimized reaction systems were utilized to determine the specificity and sensitivity of the single LAMP assay for *D. pteronyssinus* and *D. farinae*. The specificity analysis of the single LAMP assay revealed that only *D. pteronyssinus* produced an amplification curve, while no amplification was noted for the other samples (Fig. 5a). Purified genomic DNA plasmids of *D. pteronyssinus* were assayed by serial dilution of ten-fold at a known concentration. The single LAMP primer sets exhibited a limit of detection (LOD) of 40 fg/µL (Fig. 5b). The specificity analysis of the single LAMP assay for *D. farinae* revealed that only *D. farinae* presented an amplification curve, while none of the other samples exhibited amplification (Fig. 5c). The sensitivity of the single LAMP assay for *D. farinae* was determined to be 40 fg/µL (Fig. 5d).

### Specificity of the multiplex LAMP assay

**Figure 6:**
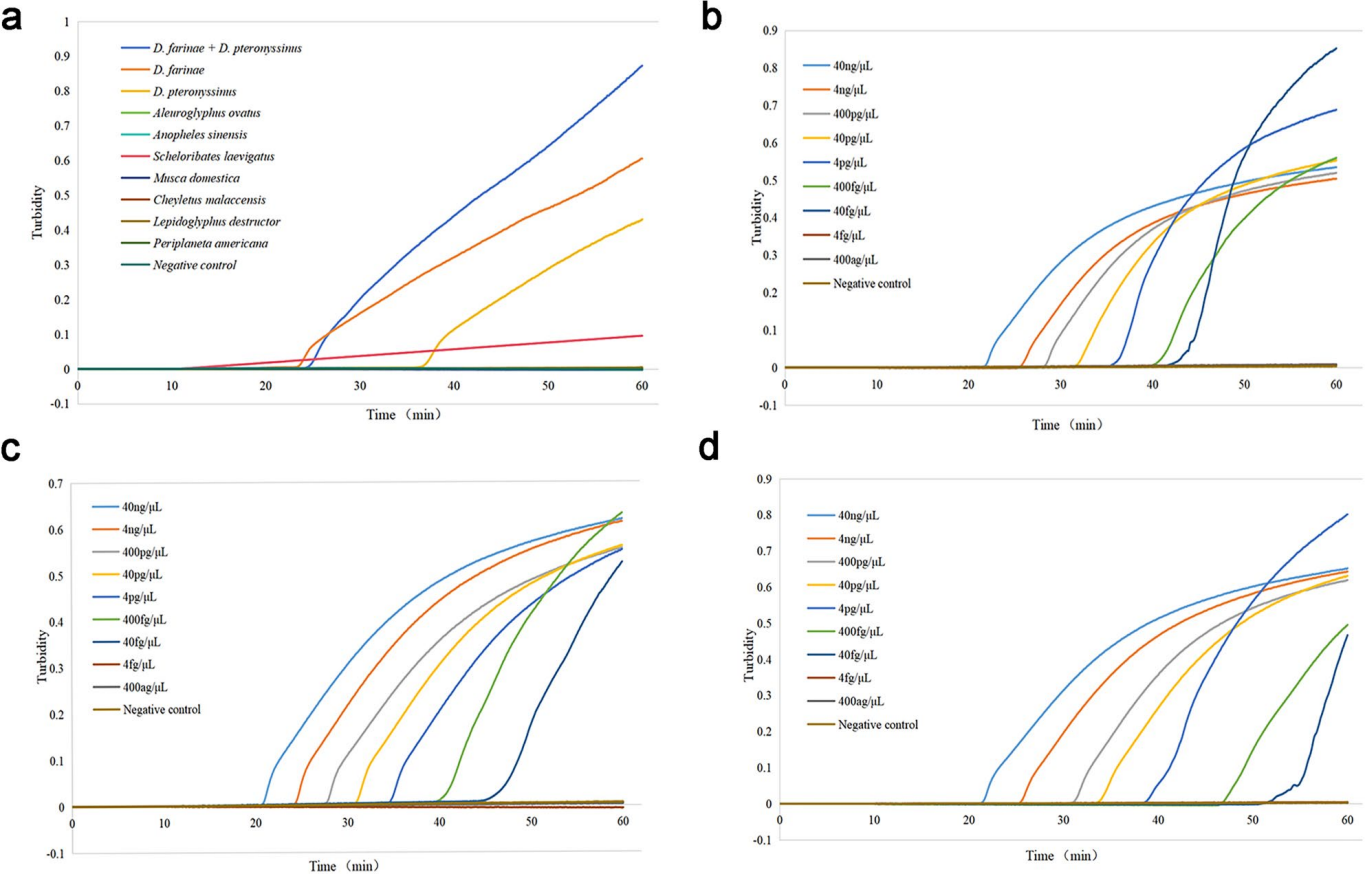
Real-time turbidity curves obtained after the multiplex LAMP assay. (**a**) Specificity of mixed ITS plasmids (*D. pteronyssinus* and *D. farinae*), *D. farinae* ITS plasmids, and *D. pteronyssinus* ITS plasmids. (**b**) Sensitivity to mixed ITS plasmids (*D. pteronyssinus* and *D. farinae*). (**c**) Sensitivity of *D. farinae* to ITS plasmids. (**d**) Sensitivity of *D. pteronyssinus* to ITS plasmids. (**b**–**d**) Dilutions within the range of 40 ng/µL to 400 ag/µL.

**Figure 7:**
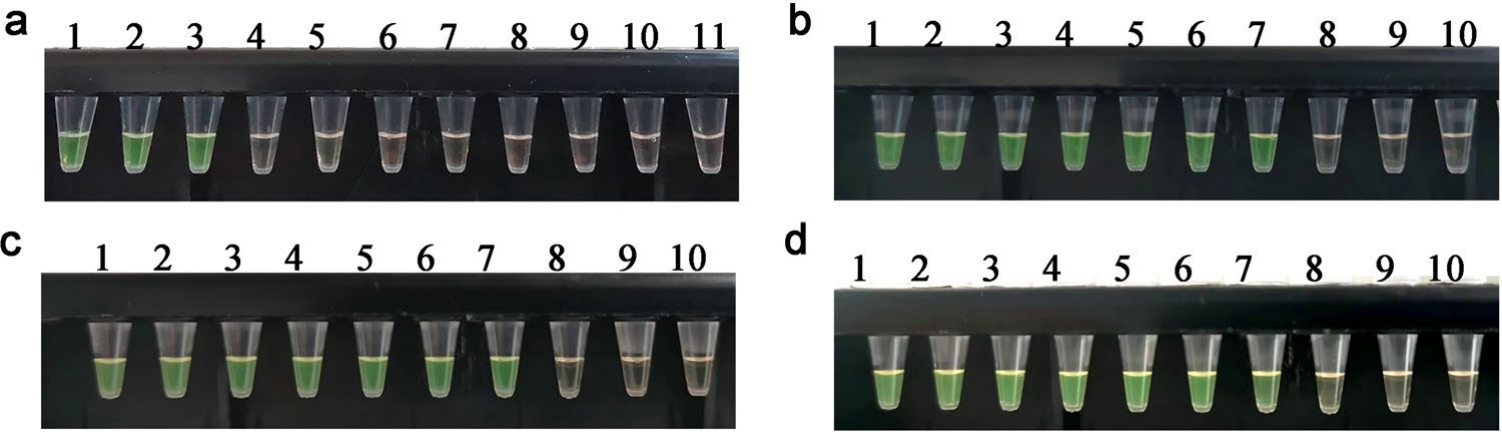
Colorimetric indication of the multiplex LAMP assay results. (**a**) Specificity of mixed ITS plasmids (*D. pteronyssinus* and *D. farinae*), *D. farinae* ITS plasmids, and *D. pteronyssinus* ITS plasmids. Samples 1–11 correspond to mixed ITS plasmids (*D. pteronyssinus* and *D. farinae*), *D. farinae*, *D. pteronyssinus*,* A. ovatus*, *An. sinensis*, *S. laevigatus*, *M. domestica*,* C. malaccensis*,* L. destructor*,* P. americana* and a negative control. (**b**) Sensitivity to mixed ITS plasmids (*D. pteronyssinus* and *D. farinae*). (**c**) Sensitivity of *D. farinae* to ITS plasmids. (**d**) Sensitivity of *D. pteronyssinus* to ITS plasmids. (**b**), (**c**), and (**d**) Samples 1–10 represent concentrations of 40 ng/µL, 4 ng/µL, 400 pg/µL, 40 pg/µL, 4 pg/µL, 400 fg/µL, 40 fg/µL, 4 fg/µL, and 400 ag/µL of the ITS plasmid, respectively, and the negative control.

**Figure 8:**
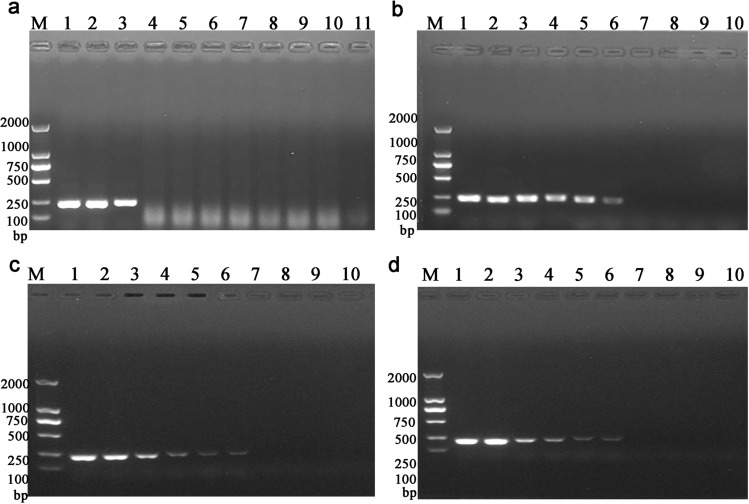
Gel electrophoresis by PCR. (**a**) Specificity of mixed ITS plasmids (*D. pteronyssinus* and *D. farinae*), *D. farinae* ITS plasmids, and *D. pteronyssinus* ITS plasmids. Lanes 1–11 represent mixed ITS plasmids (*D. pteronyssinus* and *D. farinae*), *D. farinae*, *D. pteronyssinus*,* A. ovatus*, *An. sinensis*, *S. laevigatus*, *M. domestica*,* C. malaccensis*,* L. destructor*, *P. americana* and a negative control. (**b**) Sensitivity to mixed ITS plasmids (*D. pteronyssinus* and *D. farinae*). (**c**) Sensitivity of *D. farinae* to ITS plasmids. (**d**) Sensitivity of *D. pteronyssinus* to ITS plasmids. (**b**), (**c**), and (**d**) Lanes 1–10 represent the concentrations 40 ng/µL, 4 ng/µL, 400 pg/µL, 40 pg/µL, 4 pg/µL, 400 fg/µL, 40 fg/µL, 4 fg/µL, and 400 ag/µL of the ITS plasmid, respectively, and the negative control.

Standard plasmids were utilized as templates to ensure the specificity of the multiplex LAMP assay. The results revealed that the amplification signal could be detected for the positive plasmids (*D. pteronyssinus*, *D. farinae*, *D. pteronyssinus*, *and D. farinae*) containing ITS, while all the remaining other templates were negative. In addition, the results of the FDR method were consistent with those of the turbidity assay (Figs. 6a and 7a). Subsequent comparisons with conventional PCR (Fig. 8a) also revealed bands only for the positive plasmids.

### Sensitivity of the multiplex LAMP assay

The sensitivity of the LAMP assay was evaluated, and it was revealed that the lowest detection level was 40 fg/µL for the mixed plasmids (*D. pteronyssinus* and *D. farinae*), *D. pteronyssinus* and *D. farinae* (Fig. 6b–d). The obtained turbidity curves were consistent with the findings of the FDR dye method (Fig. 7b–d). In the case of the conventional PCR method, the lowest detection level achieved was 400 fg/µL for mixed plasmids (*D. pteronyssinus* and *D. farinae*), *D. pteronyssinus* and *D. farinae* (Fig. 8b–d).

### Detection of clinical samples

Clinical sensitivity is an important criterion for assessing the usefulness of an assay. In the present study, 10 single mite clinical samples were assessed. The multiplex LAMP assay detected 40% and 70% of the single samples of the two mite species evaluated, respectively. In addition, the assay detected 80% of the mixed single mite samples. The detection validity of PCR was only 30% for *D. pteronyssinus*, 50% for *D. farinae*, and 50% for the mixed single mite samples (Table 2).

**Table 2:** Comparison of multiplex LAMP assay and PCR in single *D. pteronyssinus, D. farinae* and mixed target gene templates (*D. pteronyssinus* and *D. farinae*).

Assay	Sample		
	*D. pteronyssinus* (n = 10)	*D. farinae* (n = 10)	*D. pteronyssinus* + *D. farinae* (n = 10)
LAMP	4	7	8
PCR	3	5	5

Furthermore, 55 dust samples collected from student dormitories were evaluated. A total of 46 positive samples were detected, with a positive detection rate of 83.64% (46/55). In contrast, PCR detected only 35 positive samples and exhibited a positive detection rate of only 63.64% (35/55). The chi-squared test conducted using SPSS statistical software revealed χ^2^ = 15.687, with *P* < 0.001, indicating that the difference between the LAMP assay and PCR assay results was statistically significant (Table 3).

**Table 3:** Comparison of multiplex LAMP assay and PCR for *D. pteronyssinus and D. farinae* detection in dust samples.

PCR	LAMP		Total
	+	−	
+	35	0	35
−	11	9	20
Total	46	9	55

## Discussion

HDMs are tiny organisms that dwell on bedding and carpets and feed on skin scales shed by humans and pets. When temperature and humidity are high, dust mites survive throughout the year. *D. pteronyssinus and D. farinae* are two common species of dust mites^1,31^. Since certain people can develop allergic diseases when contact dust mites bodies and excretions, dust mites are considered to be one of the allergenic sources in humans^2,32^. Since epidemics of HDM-associated clinical diseases are associated with exposure to dust mites, it is important to be aware of the presence of HDMs inside the human household^12,30,33,34^. The accurate and efficient detection of common allergenic mites in clinical samples is essential for timely and effective control of parasitic dust mites infestation, which can mitigate the incidence of allergic diseases and improve people's quality of life standards.

The experimental results of the present study were recorded as amplification curves based on LoopAmp real-time turbidimetry and visual observation of the color change in the FDR. Next, the best primers for *D. pteronyssinus* and *D. farinae* were utilized to establish the multiplex LAMP assay. Furthermore, the optimal primers for both species were combined to optimize the development of a multiplex LAMP reaction system for the simultaneous detection of both *D. pteronyssinus* and *D. farinae*, followed by assessments of the specificity and sensitivity of this multiplex system. The findings indicated that the multiplex LAMP method had excellent specificity and presented no cross-reactivity between the multiplex LAMP primers. The LOD of the multiplex LAMP assay for both single and mixed mite samples was 40 fg/µL, which was 10 times higher than the value achieved using the PCR assay. In a previous study, Joshi et al.^29^ established a multiplex LAMP assay that achieved sensitivities of 100 fg/µL and 1 fg/µL for *Mycobacterium leprae* and *Leishmania donovani*, respectively. In China, Wang et al.^26^ successively established single LAMP and multiple LAMP assays for aquatic products of the shellfish parasites *Perkinsus* and *Bonamia*. Both *Perkinsus* and *Bonamia* presented consistent LODs (10 copies/µL for *Perkinsus* and 100 copies/µL for *Bonamia*) in both single and multiple LAMP assays. The multiplex LAMP assay developed in their study also presented good accuracy in the detection of actual samples^26^.

Clinical sensitivity is an important criterion for determining the practicality of a detection method. Thus, the multiplex LAMP assay established in this study was applied to clinical samples to determine its clinical sensitivity. Among the 10 single mite and mixed mite (*D. pteronyssinus* and *D. farinae*) samples evaluated separately, PCR detected only 3 positive samples of *D. pteronyssinus* and 5 positive samples of *D. farinae*. In addition, 5 positive samples were detected among the mixed single *D. pteronyssinus* samples and single *D. farinae* samples. On the other hand, the multiplex LAMP assay detected 4 positive samples for *D. pteronyssinus* and 7 positive samples for *D. farinae*, in addition to 8 positive samples for mixed single *D. pteronyssinus* samples and single *D. farinae* samples. In the validity test conducted with 55 clinical samples, the positive detection rate of the developed multiplex LAMP assay was 83.64%, which was significantly higher than that of PCR (63.64%). This further indicated that the developed multiplex LAMP assay had higher sensitivity and effectiveness than conventional PCR. Moreover, Fraser's group reported the presence of *Sarcoptes scabiei* DNA in several samples that were negative according to conventional PCR results. This finding corroborates that LAMP might prove to be more sensitive than the conventional PCR method for detecting *Sarcoptes scabiei*^35^. Sheng et al.^21^ demonstrated that the LAMP method was superior, more sensitive, and more effective than PCR for the detection of *Toxoplasma gondii* oocyst.

In summary, compared to conventional PCR, the multiplex LAMP assay developed in the present study offers the following advantages: (1) higher amplification efficiency; higher sensitivity, and higher specificity; (2) substantially shorter test duration; (3) simpler operation and relatively portable instrumentation, without the need for specific instruments such as agarose electrophoresis equipment and gel presenters; and (4) easy detection, visualization with the naked eye or the use of a turbidimeter that detects the precipitate to directly evaluate whether amplification occurred. In comparison, the single LAMP assay involves testing each dust mites individually in a clinical trial, which increases the gross cost and duration of diagnosis. LAMP is limited by the detection of a single target, which limits its scope and utility in real-world scenarios. Multiplex LAMP technology allows for the simultaneous detection of multiple target mites (*D. pteronyssinus* and *D. farinae*), resulting in high multiplex applicability and considerable reductions in time, cost, and effort.

The present study was the first to use the dust mites ITS region as a target gene for multiplex LAMP. Within the same set of samples, multiplex LAMP detected positive samples relatively more rapidly, accurately, and efficiently than PCR. The validity of multiplex LAMP was further confirmed with clinical samples. The present study provided strong evidence in favor of the potential of the multiplex LAMP assay in the detection of dust mites present in the indoor environment of individuals with allergy concerns. The design of effective primers is critical to the success of the LAMP assay. It is essential to use specific primers to prevent cross-reactivity with other organisms or genes.

A multiplex LAMP assay with high sensitivity and specificity for the simultaneous detection of dust mites of *D. pteronyssinus* and *D. farinae* was developed. Furthermore, *D. pteronyssinus and D. farinae* are two common species of dust mites that breed in domestic rooms. Therefore, the method established in this study is suitable for detecting the presence of dust mites dwelling in indoor living environments and is convenient for monitoring allergy-prone populations.

### Supplementary Information

Supplementary Information 1.

Supplementary Figure S1.

## Data Availability

The datasets used and/or analyzed during the current study are available from the corresponding author upon reasonable request. The ITS sequences of *D. pteronyssinus* (KC215340) and *D. farinae* (MH793957) were retrieved from the NCBI GenBank database.
