# Supplementary material for: Multiplex LAMP assay for detecting the prevalent species of dust mites Dermatophagoides farinae and Dermatophagoides pteronyssinus in the domestic environment
Source: Sci Rep. 2024 Jul 12;14:16156. doi: 10.1038/s41598-024-66043-8 (PMC11245516; doi:10.1038/s41598-024-66043-8)

Monad
莫纳生物

MonTrack™ D2000 DNA Ladder

REF：ME40601

结果展示

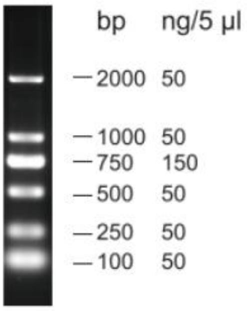

2.0% 琼脂糖凝胶电泳结果

上样量:5 μl

电泳条件:

- 电压 8 V/cm
- 1× TAE
- 电泳 30 min

Multiplex LAMP assay to detect prevalent species of dust mites: *Dermatophagoides farina* and *Dermatophagoides pteronyssinus* in the domestic environment

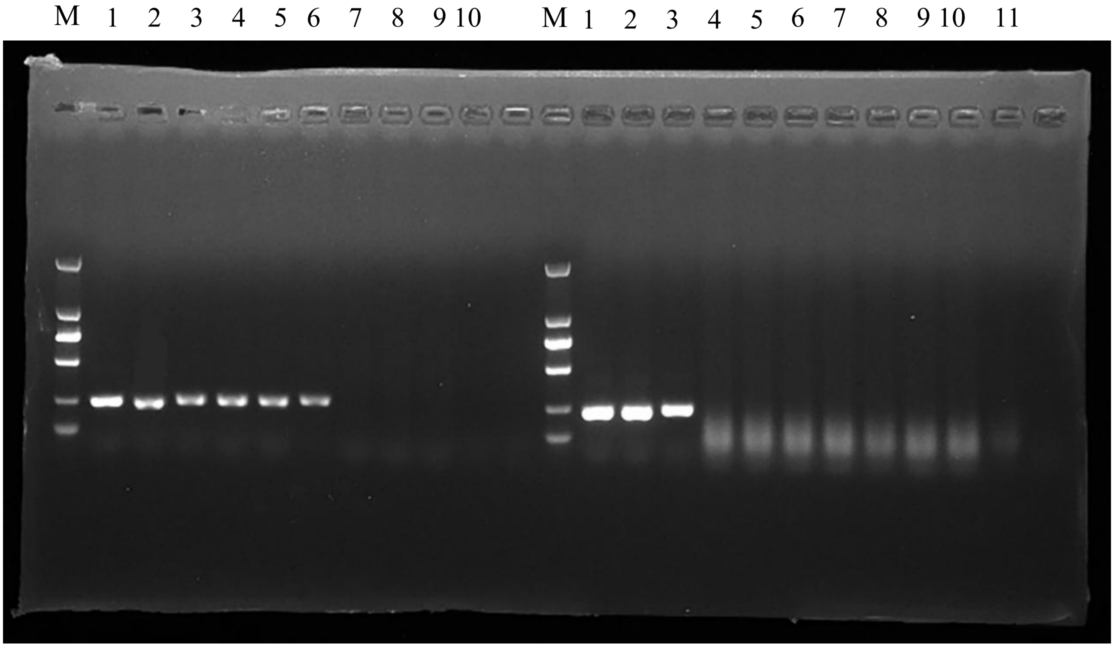

Raw image for Fig. 8b (left) and 8a (right)

After brightness/contrast adjustment

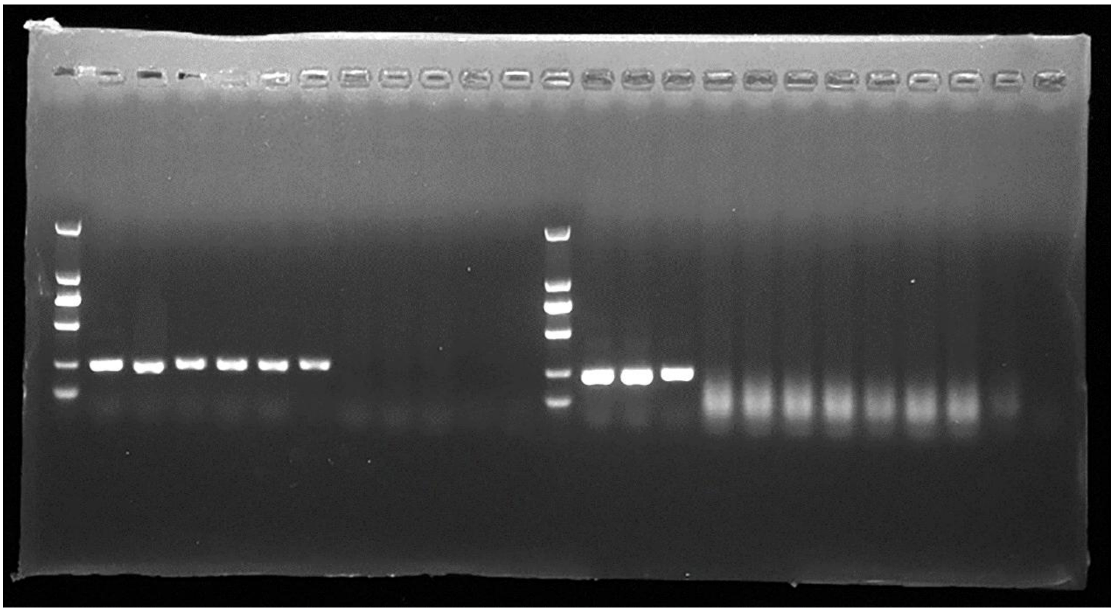

M 1 2 3 4 5 6 7 8 9 10 M 1 2 3 4 5 6 7 8 9 10

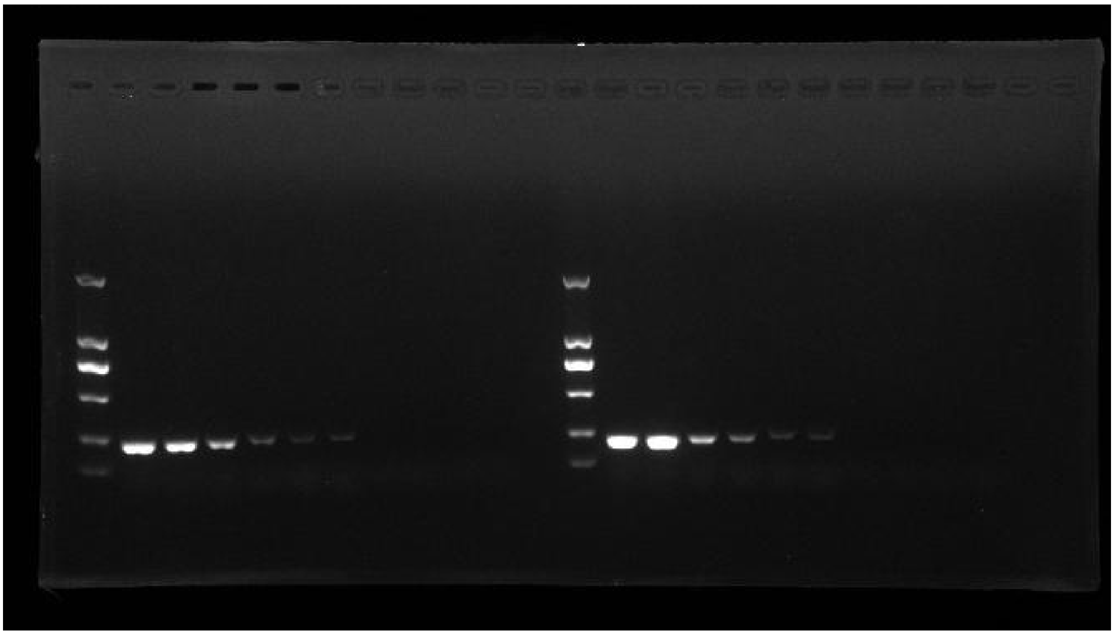

Raw image for Fig. 8c (left) and 8d (right)

After brightness/contrast adjustment

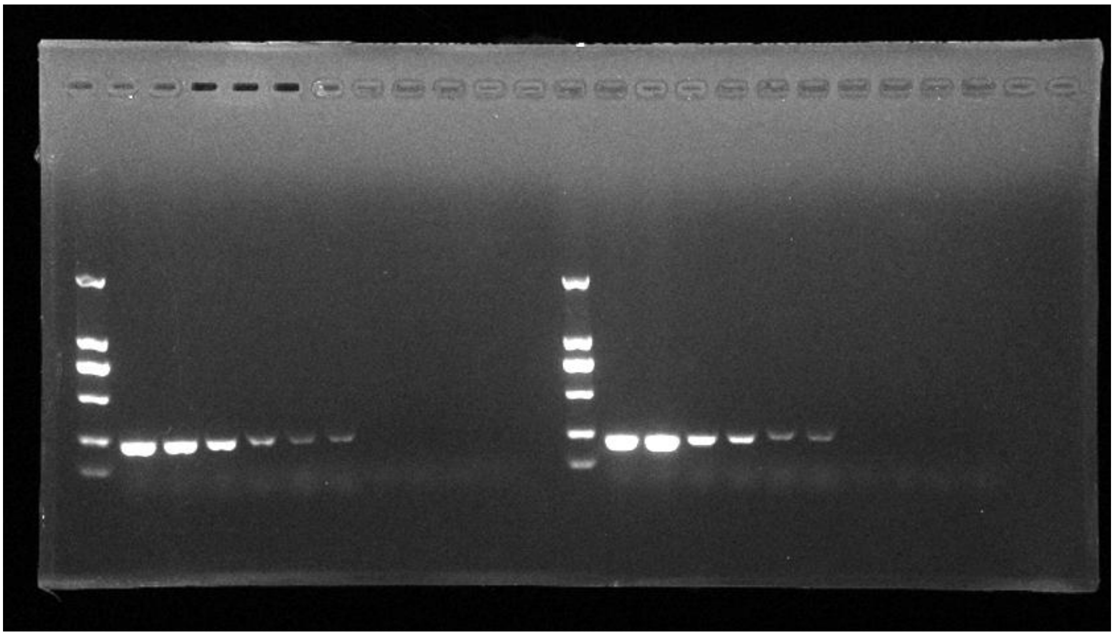

Supplement: Supplementary file 1 — Supplementary Information 1. [file 41598_2024_66043_MOESM1_ESM.pdf]
