# Supplementary material for: Multiplex LAMP assay for detecting the prevalent species of dust mites Dermatophagoides farinae and Dermatophagoides pteronyssinus in the domestic environment
Source: Sci Rep. 2024 Jul 12;14:16156. doi: 10.1038/s41598-024-66043-8 (PMC11245516; doi:10.1038/s41598-024-66043-8)
Supplement: Supplementary file 2 — Supplementary Figure S1. [file 41598_2024_66043_MOESM2_ESM.pdf]

Multiplex LAMP assay to detect prevalent species of dust mites: *Dermatophagoides farina* and *Dermatophagoides pteronyssinus* in the domestic environment

Yujun Shuai[1△] • Qiqi Xue[2△] • Huanxin Tu[1] • Junjie Guo[3] • Qiao Teng[1] •Yueye Xu[1] • Jingang Xu[1] Yuanyuan Li[1,d] • Hongming Zhou[1,4,*] • Jinhong Zhao[1,4,*]

[1] Department of Medical Parasitology, Wannan Medical College, Wuhu 241002, Anhui, China

[2] Department of laboratory medicine, Third Affiliated Hospital of Naval Medical University, Shanghai, 200438, China

[3] Department of Medical Parasitology, Qiqihaer Medical College, Qiqihaer 161000, Heilongjiang, China

[4] Anhui Province Key Laboratory of Biological Macro-molecules Research, Wannan Medical College, Wuhu 241002, Anhui, China

Corresponding author: 20100039@wnmc.edu.cn (Hongming Zhou), zhaojh@wnmc.edu.cn (Jinhong Zhao)

Supplementary material

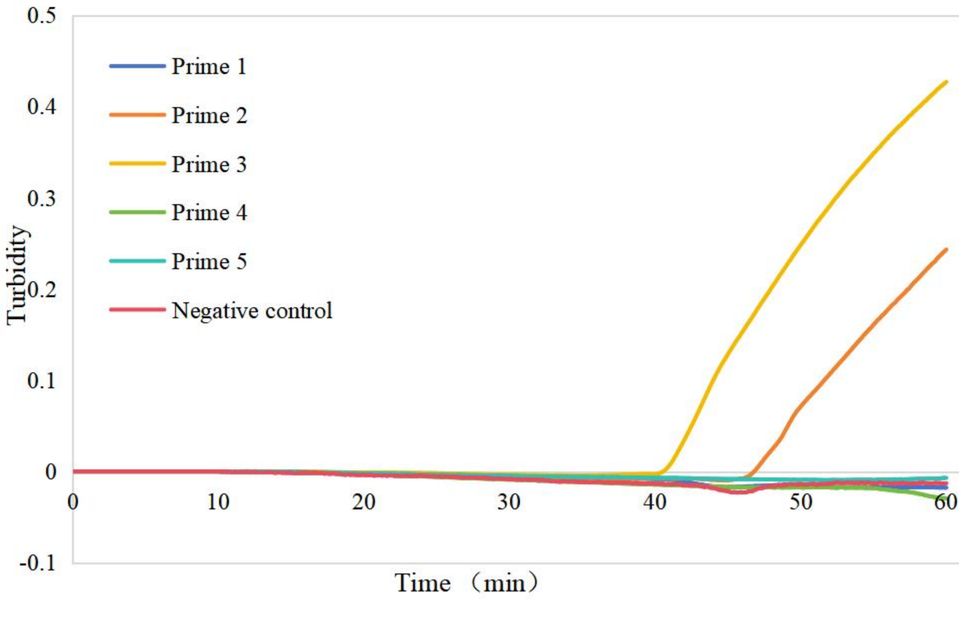

Figure S1. The 5 primer sets of *D. pteronyssinus* towards *D. farinae* for the ITS plasmid. The results showed that both primer 2 and primer 3 can amplified the dust mite *D. pteronyssinus* and *D. farinae*
